# The value of lipid accumulation products in predicting type 2 diabetes mellitus: a cross-sectional study on elderlies over 65 in Shanghai

**DOI:** 10.1007/s40200-024-01414-6

**Published:** 2024-04-18

**Authors:** Tuming Li, Shuo Yan, Dongmei Sun, Ying Wu, Huazheng Liang, Qinghu Zheng, Ping Zhong

**Affiliations:** 1 Department of Neurology, Shidong Hospital, 999 Shiguang Road, Yangpu District, Shanghai, 200438 China; 2 Shanghai Medical College of Integrated Traditional Chinese and Western Medicine, Shanghai University of Traditional Chinese Medicine, Shanghai, China; 3 Henan University of Traditional Chinese Medicine, Henan, China; 4 Community Health Service Center, Pudong New Area, Shanghai, China; 5 Clinical Research Center for Anesthesiology and Perioperative Medicine, Shanghai Fourth People’s Hospital, School of Medicine, Tongji University, Shanghai, China; 6 Monash Suzhou Research Institute, Suzhou Industrial Park, Suzhou, Jiangsu Province China

**Keywords:** Lipid accumulation product, Type 2 diabetes mellitus, Risk model, Correlation analysis

## Abstract

**Purpose:**

As lifestyle changes, there is an increasing number of type 2 diabetes mellitus (T2DM) patients in China. The present study aimed to investigate the predictive value of the lipid accumulation product (LAP) for T2DM in Chinese elderlies over 65 years.

**Methods:**

The present cross-sectional study recruited 2,092 adults from communities of Pudong New Area of Shanghai. Questionnaires were filled and anthropometric and laboratory examinations were completed by all participants. The predictive value of different risk factors for T2DM was analyzed using the receiver operating characteristics curve (ROC).

**Results:**

LAP was found to be closely related to T2DM (adjusted OR: 0.613, 95% CI: 0.581–0.645). Fasting plasma glucose (FPG), LAP, and urea nigrogen (UN) were associated with T2DM in females, whereas FPG, LAP, neck circumference (NC) were associated with T2DM in males. When the cut-off value was 33.8, LAP displayed the optimal predictive performance. A gender difference was observed with an LAP of 37.95 demonstrating the best predictive value in males (AUC = 0.604, 95% CI: 0.577–0.652) and 60.2 in females (AUC = 0.617, 95% CI: 0.574–0.660), respectively.

**Conclusion:**

LAP is more significantly associated with the risk of T2DM in elderlies than FPG, UN or NC, and it serves as a strong predictor of T2DM. However, this is impacted by FPG and neck circumference to a certain extent. Future large-scale studies are needed to confirm its efficacy in predicting diabetes.

## Introduction

Type 2 diabetes mellitus (T2DM), one of the leading non-communicable diseases in the middle-aged population worldwide, is largely attributed to recent changes in diets and lifestyles [1, 2]. As estimated by the International Diabetes Federation in 2017, approximately 425 million people suffered from diabetes in the world and China had nearly 114 million patients, ranking first in the world [3]. This indicates that 10.91% of the Chinese population has diabetes, which is an emerging challenge for China. Effective measures to predict the risk of developing diabetes and early intervention that targets the risk factors can significantly reduce the incidence of T2DM [4]. Obesity is one such independent risk factor for diabetes. Epidemiological data have shown that the impact of obesity on the level of blood glucose is not only associated with the content of fat but also with the distribution pattern of fat [5]. Currently, there are a few commonly used anthropometric methods to assess obesity, including BMI, waist circumference (WC), waist-to-hip ratio and others. These methods are simple and convenient to complete, but there are certain limitations in clinical practice [6]. Lipid accumulation product (LAP), proposed by American researchers based on results from the third National Health and Nutrition Examination Survey of the United States, is considered to be a better index to assess obesity [7]. Compared with traditional indicators, LAP takes the waist circumference and the level of blood triglyceride (TG) into consideration. The former reflects the extent of fat accumulation in the abdomen and the latter is associated with the extent of fat accumulation in internal organs [8]. LAP was calculated as (WC − 60.6)×(TG [mmol/L]) in males and (WC − 54.1)×(TG [mmol/L]) in females as proposed by a Chinese population study in Shanghai. Furthermore, LAP was reported to be closely related to cardiovascular diseases [9], hypertension [10], and metabolic syndrome [11]. However, little is known about the correlation of LAP with T2DM. The present study aimed to answer this question by completing a cross-sectional epidemiological investigation in communities in Shanghai.

## Materials and methods

### Subjects

The present study recruited 2,092 participants from 11 communities in Pudong New Area of Shanghai between January and March 2012. Our inclusion criteria were: participants lived in corresponding communities for at least 5 years; their age was 65 or older, they voluntarily participated in this study. The exclusion criteria were: participants were inflicted by thyromegaly or other systemic medical diseases (like kidney and liver dysfunction, heart failure, AIDS, cancer, etc.); participants were professional or amateur athletes, bodybuilders, etc. Written informed consent was obtained from all participants before the start of this study.

### Collection of clinical information

Demographical and clinical information was collected using a questionnaire including gender, age, alcohol consumption, smoking status, frequency of physical exercise, history of hypertension, diabetes, and coronary heart diseases. Current smokers refers to individuals who smoked every day or every few days and consumed at least 100 cigarettes in his/her lifetime. Alcohol drinking refers to daily drinking of 25 g including beer 750 ml/wine 250 ml/Baijiu (53^o^) 50 ml (38^o^) 75 ml or more alcohol-containing beverage for males and at least 15 g for females including beer 450 ml/wine 150 ml/Baijiu (53^o^) 30 ml (38^o^) 50 ml. History of hypertension was confirmed by the presence of systolic blood pressure (SBP) ≥ 140 mmHg or diastolic blood pressure (DBP) ≥ 90 mmHg 3 times in 3 days (once a day) or a time period of taking anti-hypertensive drugs. Coronary heart diseases (CHD) refer to the presence of atherosclerosis blocking up to 50% or more of the coronary artery on angiography or a positive history of acute myocardial infarction. Dyslipidemia refers to a level of total cholesterol (TC ≥ 5.2 mmol/L) and/or triglycerides (TG ≥ 1.7 mmol/L) above the normal range. Diabetes refers to a high level of fasting plasma glucose (FPG ≥ 7 mmol/L), or a positive history of taking antidiabetic drugs or insulin injection. Urea nitrogen was available from the routine kidney function test.

### Anthropometry

Participants were in light dresses and bear-footed for their measurement of body height and weight. Measurements were repeated twice with a 10-minute interval and averaged. The waist circumference (WC) was measured in the zone between the crista iliac and the lower rib margin. The horizontal neck circumference (NC) was measured at the upper margin of the laryngeal prominence while keeping their heads straight and their eyes looking forward. The body mass index (BMI) was calculated through dividing the body weight (kg) by the square of height (m).

### Blood biochemical detections

Fasting blood samples were obtained from each participant and processed in the laboratory to test the levels of FPG, TC, TG, and the total bilirubin (TBIL). These samples were stored in a -80 °C deep freezer before testing.

### Statistical analysis

In the present study, quantitative data were expressed either as the mean ± standard deviation (SD) or the median/interquartile range, and qualitative data were expressed as ratios. Quantitative data were tested using the Kolmogorov-Smimov test to evaluate whether they were normally distributed or not, which determined the method for statistical analysis. The difference in the quantitative data between different groups was analyzed using the Kruskal-Wallis H test when these data were not normally distributed. In contrast, the difference in the quantitative data between groups was analyzed using the unpaired *t* test if they were normally distributed. Difference in categorical data was analyzed by adopting the Chi-square test. The logistic stepwise regression was adopted to examine risk factors with significant differences for T2DM. The binary logistic regression analysis was used to assess the impact of risk factors on T2DM. The value of LAP was divided into Q1, Q2, Q3 and Q4 and its correlation with T2DM was explored. The Spearman correlation analysis was then used to evaluate the correlation between LAP and other risk factors of T2DM. The receiver operating characteristic (ROC) curve was applied to assess the predicting capacity of individual risk factors on T2DM. *P* < 0.05 indicated statistically significant difference. Statistical analyses were conducted with the assistance of the SPSS software (version 26.0, SPSS Inc., Chicago, IL, USA).

## Results

### Demographic characteristics of study participants

The present study recruited 2,092 participants with an average age of 73.21 ± 6.711 years. Among them, 971 were males and 1,121 were females. Among them, 1,746 had a history of T2DM and 346 had no history of T2DM. There was no significant difference in the history of T2DM between males and females. Differences in hypertension (*P* < 0.001) and gender (*P* < 0.001) were observed between groups with and without T2DM. In contrast, no statistically significant difference was found in age, smoking, alcohol consumption, or history of coronary heart diseases between groups with and without T2DM. A statistically significant difference was also found in body weight (*P* < 0.001), waist circumference (*P* < 0.001), neck circumference (*P* < 0.001), BMI (*P* < 0.001) and systolic blood pressure (*P* < 0.001) between groups with or without a history of T2DM and between groups of different genders. A significant difference was observed in laboratory tests, such as FPG (*P* < 0.001), TG (*P* < 0.001) and LAP (*P* < 0.001), between groups with and without a history of T2DM. The gender difference was also seen in these tests (FPG: *P* < 0.001; TG: *P* < 0.001; LAP: *P* < 0.001)(shown in Tables 1 and 2).

### Screening of factors related to type 2 diabetes and gender differences of related factors

Spearman correlation analysis showed that body weight (*P* < 0.001), neck circumference (*P* < 0.001), systolic blood pressure (*P* < 0.001), FPG (*P* < 0.001), LAP (*P* < 0.001) and BMI (*P* < 0.001) were all significantly associated with T2DM. Detailed results are shown in Table 3. Stepwise regression analysis demonstrated that FPG (*P* < 0.01) and NC (*P* = 0.019) were associated with T2DM in the study population. However, only FPG (*P* < 0.01) and NC (*P* < 0.001) were significantly associated with the history of T2DM in the male population. In contrast, only FPG (*P* < 0.01) was significantly associated with the history of T2DM in the female population (Table 4).

### Analysis of related indicators and risk factors for T2DM

FPG (*P* < 0.001) and LAP(*P* < 0.001) were significantly associated with the risk of T2DM before and after adjusting for age, sex, smoking, alcohol consumption, and other factors. This relationship between FPG (*P* < 0.001) and the risk of T2DM remained after adjusting associated indices of lipids and blood pressure, whereas no correlation was found between the risk of T2DM and NC (*P* > 0.05), nor between T2DM and LAP (*P* > 0.05).

With Q1 as the reference, before and after adjusting these parameters for age, gender, smoking, alcohol consumption and other factors, NC (before: OR = 2.17; after: OR = 2.5), FPG (before: OR = 1.94; after: OR = 1.94), LAP (before: OR = 1.97; after: OR = 1.97) were significantly associated with the risk of T2DM for patients with their LAP in Q4 (*P* < 0.001). After adjusting for lipid and blood pressure associated indices, LAP (*P* > 0.05) was not significantly associated with the risk of T2DM, but NC (*P* < 0.001) and FPG (*P* < 0.001) were.

For males, NC (*P* < 0.001), FPG (*P* < 0.001) and LAP (*P* < 0.001) were significantly associated with the risk of T2DM before and after adjusting for age, sex, smoking, alcohol consumption, and other factors. FPG (*P* < 0.001) was significantly related to the risk of T2DM after adjusting lipids and blood pressure-related indices, but NC and LAP (*P* > 0.05) were not. With Q1 as the reference, before and after adjusting these parameters for age, gender, smoking, alcohol consumption and other factors, NC (before: OR = 3.24; after: OR = 2.5), FPG (before: OR = 1.94; after: OR = 1.94), LAP (before: OR = 1.97; after: OR = 1.97) were significantly associated with the risk of T2DM for patients with their LAP in Q4 (*P* < 0.001). After adjusting for lipid and blood pressure associated indices, LAP (*P* > 0.05) and NC (*P* > 0.05) were not significantly associated with the risk of T2DM, but FPG (*P* < 0.001) was.

For females, NC (*P* < 0.001), FPG (*P* < 0.001), and LAP (*P* < 0.001) were significantly associated with the risk of T2DM before adjusting for age, sex, smoking, alcohol use, and only FPG (*P* < 0.001) and LAP (*P* < 0.001) were associated with the risk of T2DM after adjusting for these factors.

When Q1 was set as the reference, NC, FPG, and LAP before [NC (OR = 3.97), FPG (OR = 22.53), LAP (OR = 2.06)] and after [NC (OR = 4.04), FPG (OR = 22.8), LAP (OR = 2.08)] adjusting for age, sex, smoking, alcohol consumption and other factors were significantly associated with the risk of T2DM for patients with their LAP in Q4 (*P* < 0.001). NC (*P* < 0.05) and FPG (*P* < 0.001) were significantly associated with the risk of T2DM after adjusting for lipid and blood pressure-associated indices, whereas LAP (*P* > 0.05) was not. These suggest that there is a stronger correlation between the above factors and the risk of T2DM when the value of LAP is higher in either the overall population or the male or female population. In addition, LAP was influenced by several confounding factors (Table 5).

### Confounding factor analysis of NC, FPG, and LAP

Logistic regression analysis was conducted to test possible factors that impact NC, FPG, and LAP, aiming to find confounding factors associated with LAP. Univariate analysis showed that NC [OR = 1.11(95%CI 1.07–1.15) *P* < 0.001], FPG [OR = 2.2 (95%CI 2.01–2.42) *P* < 0.001], LAP [OR = 1.01 (95%CI 1.007–1.012) *P* < 0.001] showed statistically significant difference. Multivariate Logistic regression analysis showed that FPG significantly impacted LAP of 3 groups [OR = 1.001 (95%CI 0.998–1.005) (overall), OR = 0.998 (95%CI 0.993–1.004)(male), OR = 1.003 (95%CI 0.99–1.008)(female)] after taking NC, FPG, LAP, BMI and TG into consideration. In the male population, FPG significantly influenced NC [OR = 1.05 (95%CI 0.97–1.13) *P* > 0.05], whereas NC influenced LAP [OR = 1.003 (95%CI 0.99–1.008) *P* > 0.05] in the female population (Table 6).

### Prediction of T2DM using ROC curves of related factors

**Fig. 1:**
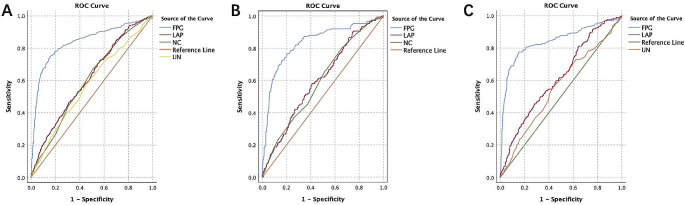
Prediction of T2DM using the ROC curves of associated indices. Note: FPG: fasting plasma glucose; LAP: lipid accumulation product; NC: Neck circumference; UN: urea nitrogen

For the general population, FPG had the largest AUC (0.839, 0.812–0.866) and it showed the best predicting effect at the concentration of 6.4 mmol/L. LAP had the second largest AUC (0.613, 0.581–0.645) and the best predicting effect at 33.8 mmol/L. NC had an AUC of 0.6 (0.569 − 0.532) and the best prediction effect at 35.75 cm. For males, FPG had the best predicting effect (AUC = 0.835, 0.795–0.874) at 6.49 mmol/L, followed by LAP (AUC = 0.604, 0.577-652) at 37.95 mmol/L and NC (AUC = 0.597, 0.594–0.644) at 36.9 cm. For females, FPG was also the best predictor (AUC = 0.842, 0.805–0.878) at 6.42 mmol/L, followed by LAP (AUC = 0.617, 0.574–0.660) at 60.2 mmol/L (Table 7; Fig. 1).

## Discussion

The present study investigated factors related to T2DM in a community population. It was found that LAP was closely related to T2DM. FPG, LAP and NC were all associated with T2DM with slight gender differences. FPG and LAP were the leading factors that can predict T2DM with FPG being the most powerful one.

A sedentary lifestyle and high-calorie diets significantly increase the risk of obesity and the occurrence and development of diabetes [12]. Studies have shown that abdominal obesity is more closely associated with cardiovascular diseases, diabetes and other metabolic diseases compared with systemic obesity. Visceral adipose tissue is one of the key sources of various proinflammatory cytokines that target the liver. They are more influential than other types of adipose tissue on metabolism-related disorders by undermining the signal transduction of insulin, resulting in insulin resistance, lipid accumulation, and altered metabolism [13–15]. It has been shown that high levels of visceral fat are an independent risk factor for T2DM [16].

Currently, there are two commonly used methods to measure visceral fat, imaging detection and anthropometric detection. Imaging methods mainly include CT and MRI, which have the advantages of high accuracy and good repeatability. However, they are relatively expensive and patients need to be exposed to X-rays for CT detection. Therefore, they are not suitable for promotion and application in the clinic. Although BMI, NC and obesity have been increasingly used in anti-diabetic medication, they all have their relative limitations [17]. The body mass index couldn’t reflect fat distribution, there are still many people with excessive visceral fat but normal BMI. Neck circumference as another conventional index has been positively associated with visceral fat area (VFA) [17]. Some studies show that visceral fat (especially in the liver) is closely related to insulin resistance, as well as been associated with the prevalence of T2DM and prediabetes in obese individuals [18]. Accurate quantification of fat content in the liver can predict the occurrence and future development of T2DM in patients [19]. Subjects with excessive visceral adipose tissue (VAT), regardless of their BMI or overall obesity, have a higher risk of T2DM and worse cardiovascular outcomes [18]. LAP is based on the combination of WC and TG and is a good indicator of visceral and abdominal fat accumulation. It has been adopted by a growing number of researchers [5]. Commonly used LAP is calculated based on the data of European and American populations, which might be different from that of individual Chinese populations. Therefore, the present study calculated LAP using the modified formula proposed by Junxuan Huang to reflect the real data of fat accumulation in internal organs and the abdomen of Chinese populations. Compared to BMI, LAP mainly measures visceral fat, which plays an important role in the development of chronic low-grade inflammation and insulin resistance, and ultimately affects the development of T2DM. LAP was first proposed by Kahn in 2015 because of its superiority over BMI in the identification of cardiovascular risks [7]. In a recent study, LAP has the potential to be considered as a predictor of T2DM apart from cardiovascular diseases [20]. In recent years, LAP has been more presented in T2DM studies, a systematic review and meta-analysis published in 2022 showed that most researches present a result that LAP is more advantageous in predicting T2DM compared to conventional indices including BMI and obesity [21], and LAP has a stronger correlation with insulin resistance compared to BMI, and the degree of insulin resistance increases with the increase of LAP [22]. Some studies about T2DM in Asia even didn’t enlist LAP for the researchers [23].

In the present study, logistic regression analysis showed that LAP was significantly associated with the risk of T2DM with good predicting power. In the corrected regression analysis, confounding factors were found to be responsible for the negative LAP results, and further multivariate regression analysis showed that FPG was the culprit. Studies have shown that FPG has a sustained effect on LAP, and the fluctuating FPG can elicit changes in many obesity-related indicators, including LAP, with gender differences between subjects with or without T2DM [24]. A 7.77-year follow-up cohort study showed that both LAP and high triglyceride waist circumference (HTGW) were associated with increased risk of T2DM, and there was a non-linear correlation between LAP and T2DM, and HTGW, and interaction between high LAP, high FPG and the risk of T2DM [25].

ROC curve results showed that the diagnostic prediction ability of LAP was inferior to that of FPG. This might be explained by the nature of this study being a cross-sectional one and the majority of patients with diabetes had impaired fasting glucose. The correlation between diabetes and impaired fasting glucose was better than that between diabetes and LAP. Clinically, for subjects without impaired fasting glucose, there is no direct correlation between the level of fasting glucose and the occurrence of diabetes. However, if subjects are obese, they are more likely to have a higher risk of T2DM than non-obese subjects based on findings of an epidemiological study on obesity and T2DM [5]. Therefore, it is not confirmative that the ROC result of LAP was inferior to FPG in predicting T2DM due to the nature of the present study.

The CUT-off value of this study was 33.8 (sensitivity, 72%; specificity, 44%) in the study population, and the AUC was 0.613, which is similar to results of a couple of studies. Shu et al. (2022) reported a cut-off value of 33.54 with an AUC of 0.650 (sensitivity, 54.6%; specificity, 62.42%) [26]. This study was conducted in Bengbu, Anhui Province of Eastern China. The other study in Urumqi on a population of 215,651 adults, it was found that LAP was better than BMI in predicting cardiovascular risk and the predictive accuracy was better than BMI when the cut-off value was 38.045 (sensitivity = 60.6%, specificity = 62.0%) [27].

In the female population of the present study, NC, a marker of subcutaneous fat deposition in the upper part of the body, was shown to be a confounding factor of LAP. LAP, calculated using WC and TG, reflects fat deposition in the abdomen and internal organs [28]. It has been reported that fat deposition in the upper body (mainly focused on the abdomen) of females is higher than that of males [29]. This might account for the difference in the prediction of and correlation of LAP with diabetes between different genders and the influence on the statistical results of LAP.

There are some limitations in the present study. Firstly, the small sample size of this study can not pinpoint the causal relationship between T2DM and the predictors. The population was confined to elderlies in Shanghai, and there were gender differences in predicting T2DM using LAP and the risk correlation of LAP with T2DM. More studies are needed to determine whether predicting the risk of T2DM using LAP can be applied to all Chinese populations.

## Conclusion

LAP is a good indicator of visceral fat deposition and it is more significantly associated with the risk of T2DM in elderlies than FPG and NC or UN. It can be used as a good diagnostic predictor of T2DM, but is influenced to a certain extent by FPG and NC. Future large-scale studies are needed to confirm its efficacy in predicting diabetes.

**Table 1:** Baseline characteristics of subjects

Variables	N (*N* = 1746)	2-DM(*N* = 346)	X^2^/Z	P value
Sex (%)			1.5631	0.2112
Male	47.02%	43.35%		
Female	52.98%	56.65%		
Age (years)	73.139 ± 6.800	73.549 ± 6.234	−1.04	0.2986
Smoker (%)	14.49	11.27	2.4909	0.1145
Drinker (%)	16.27	13.58	1.5595	0.2117
History of CHD(%)	15.64	19.36	2.9493	0.0859
History of hypertension	47.08	70.23	61.936	< 0.001^***^
Height	160.10 ± 8.437	160.47 ± 8.324	−0.74	0.4595
Weight	60.559 ± 10.963	64.13 ± 10.178	−5.60	< 0.001^***^
NC	35.771 ± 3.278	36.911 ± 3.028	−5.98	< 0.001^***^
BMI	23.572 ± 3.5773	24.871 ± 3.298	−6.25	< 0.001^***^
SBP	134.07 ± 15.907	137.45 ± 15.78	−3.62	< 0.001^***^
DBP	77.906 ± 8.7723	77.939 ± 8.4948	−0.06	0.9484
FPG	5.8907 ± 1.1058	8.2143 ± 2.6805	−26.58	< 0.001^***^
TG	1.5047 ± 0.8566	1.783 ± 1.1353	−5.21	< 0.001^***^
LAP	39.099 ± 29.595	53.144 ± 44.694	−7.33	< 0.001^***^

*Notes* LAP: Lipid accumulation product; WC: Waist circumference; NC: Neck circumference; WHR: Weight-height ratio; WNR: Weight-neck circumference ratio; CHD: Coronary heart disease; HBP: High blood pressure; TG: Triglyceride; TC: Total cholesterol; FPG: Fasting plasma glucose; TBIL: Total bilirubin; SBP: Systolic blood pressure; DBP: Diastolic blood pressure; CHD: coronary heart disease. P value < 0.05 was considered statistically significant. ^***^: *P* < 0.001

**Table 2:** Baseline characteristics of subjects

Vatiables	Male(*N* = 971)		P value	Female(*N* = 1121)		P value
	NO	2-DM		NO	2-DM	
Age (years)	72.677 ± 6.440	73.167 ± 5.832	*P* > 0.05	73.548 ± 7.0832	73.842 ± 6.5242	*P* > 0.05
Smoker (%)	26.80%	2.67%	*P* > 0.05	3.57%	2.55%	*P* > 0.05
Drinker (%)	29.60%	28.00%	*P* > 0.05	4.43%	2.55%	*P* > 0.05
CHD (%)	14.01%	19.33%	*P* > 0.05	17.08%	19.39%	*P* > 0.05
Hypertension (%)	46.77%	63.33%	*P* < 0.001^***^	47.35%	75.51%	*P* < 0.001^***^
Height	166.86 ± 6.045	167.20 ± 6.131	*P* > 0.05	154.47 ± 5.8776	155.32 ± 5.6902	*P* > 0.05
Weight	65.219 ± 10.433	68.613 ± 9.104	*P* < 0.001^***^	56.423 ± 9.6935	60.699 ± 9.6255	*P* < 0.001^***^
BMI	23.513 ± 3.405	24.519 ± 2.777	*P* < 0.001^***^	23.623 ± 3.7243	25.141 ± 3.6304	*P* < 0.001^***^
NC	37.657 ± 2.814	38.637 ± 2.632	*P* < 0.001^***^	34.096 ± 2.7022	35.59 ± 2.6241	*P* < 0.001^***^
SBP	133.27 ± 16.138	136.15 ± 15.711	*P* < 0.05^*^	134.78 ± 15.674	138.45 ± 15.80	*P* < 0.01^**^
DBP	78.527 ± 8.81	78.74 ± 9.266	*P* > 0.05	77.355 ± 8.7022	77.327 ± 7.8227	*P* > 0.05
FPG	5.899 ± 1.092	8.054 ± 2.445	*P* < 0.001^***^	5.8829 ± 1.118	8.3367 ± 2.8474	*P* < 0.001^***^
TG	1.4133 ± 0.8269	1.7007 ± 1.2285	*P* < 0.001^***^	1.5859 ± 0.8746	1.846 ± 1.0573	*P* < 0.001^***^
LAP	33.787 ± 27.137	44.829 ± 38.864	*P* < 0.001^***^	43.814 ± 30.875	59.507 ± 47.813	*P* < 0.001^***^

*Notes* LAP: Lipid accumulation product; NC: Neck circumference; CHD: Coronary heart disease; TG: Triglyceride; FPG: Fasting plasma glucose; SBP: Systolic blood pressure; DBP: Diastolic blood pressure; CHD: coronary heart disease. P value < 0.05 was considered statistically significant. ^**^: *P* < 0.01; ^***^: *P* < 0.001

**Table 3:** Correlations between T2DM and related factors

	Weight	NC	FPG	TG	LAP	BMI
Z	−5.687	−5.934	−19.942	−5.061	−6.652	−6.368
*P*	< 0.001^***^	< 0.001^***^	< 0.001^***^	< 0.001^***^	< 0.001^***^	< 0.001^***^

*Notes* Number means Correlation Coefficient; LAP: lipid accumulation product; BMI: Body mass index; FPG: fasting plasma glucose; TG: Triglyceride; NC: Neck circumference; ^***^: *P* < 0.001; P means P value

**Table 4:** Results of stepwise regression analysis of factors associated with T2DM

Variables	OR	95%C.I. for OR	P value
Overall			
NC	1.053	1.008–1.099	< 0.05^*^
FPG	2.16	1.996–2.374	< 0.001^***^
Men			
FPG	2.130	1.866–2.430	< 0.001^***^
NC	1.275	1.119–1.453	< 0.001^***^
Female			
FPG	2.173	1.905–2.480	< 0.001^***^

*Notes* LAP, lipid accumulation product; NC: Neck circumference; FPG: fasting plasma glucose; BMI: Body mass index; ^*^: *P* < 0.05; ^***^: *P* < 0.001

**Table 5:** Results of regression analysis of factors associated with T2DM

Variables	OR(95%CI)†			OR(95%CI)‡			OR(95%CI)§		
	NC	FPG	LAP	NC	FPG	LAP	NC	FPG	LAP
*Overall*									
Total	1.11^***^	2.20^***^	1.01^***^	1.13^***^	2.21^***^	1.01^***^	1.04	2.14^***^	1.01
Q1	1(ref.)	1(ref.)	1(ref.)	1(ref.)	1(ref.)	1(ref.)	1(ref.)	1(ref.)	1(ref.)
Q2	1.63^***^	0.88	1.81	1.71^**^	0.87	1.79	1.4	0.86	1.52
Q3	2.13^***^	1.83^*^	1.83	2.3^***^	1.82^*^	1.82	1.5	1.73^*^	1.25
Q4	2.17^***^	1.94^***^	1.97^***^	2.5^***^	1.94^***^	1.97^***^	1.3^***^	17.72^***^	1.04
*Men*									
Total	1.13^***^	2.11^***^	1.01^***^	1.14^***^	2.15^***^	1.01^***^	1.09	2.14^***^	0.98
Q1	1(ref.)	1(ref.)	1(ref.)	1(ref.)	1(ref.)	1(ref.)	1(ref.)	1(ref.)	1(ref.)
Q2	1.86	0.5	1.01	1.97	0.5	1.01	1.53	0.5	1.19
Q3	3.2^**^	2.31^*^	0.68	3.37^**^	2.26^*^	0.67	1.94	2.23^*^	0.48
Q4	3.24^**^	16.35^***^	1.91^***^	3.43^**^	16.41^***^	1.98^***^	1.93	15.72^***^	1.09
*Women*									
Total	1.22^***^	2.3^***^	1.01^***^	1.23^***^	2.3^***^	1.01^***^	1.14^**^	2.18^***^	1.01
Q1	1(ref.)	1(ref.)	1(ref.)	1(ref.)	1(ref.)	1(ref.)	1(ref.)	1(ref.)	1(ref.)
Q2	1.93^**^	1.23	2.61^*^	1.92^**^	1.22	2.59^*^	1.61	1.2	1.53
Q3	2.59^***^	1.48	3.71^**^	2.62^***^	1.5	3.69^**^	1.68	1.33	2.09
Q4	3.97^***^	22.53^***^	2.06^***^	4.04^***^	22.8^***^	2.08^***^	2.2^*^	19.57^***^	0.8

*Notes* LAP, lipid accumulation product; NC: Neck circumference; FPG: fasting plasma glucose; BMI: Body mass index; *: *P* < 0.05;**: *P* < 0.01;***: *P* < 0.001; †:before adjustment; ‡:after adjusting for age, gender, smoking, alcohol drinking;§: after adjusting for lipid (LAP/BMI/FPG/TG/NC) and blood pressure (SBP/DBP/HYP) indices

**Table 6:** Logistic regression analysis of confounding factors and verification

Variables	OR (95%CI) †	OR(95%CI) ∫				
		NC	FPG	LAP	BMI	TG
*Overall*						
NC	1.11 (1.07–1.15)^***^	–	1.05 (1.01–1.1)^*^	1.08 (1.04–1.12)^***^	1.07 (1.02–1.12)^**^	1.1 (1.06–1.14)^***^
FPG	2.2 (2.01–2.42) ^***^	2.16 (1.96–2.37)^***^	–	2.17 (1.98–2.4)^***^	2.16 (1.96–2.37)^***^	2.2 (2.003–2.42)^***^
LAP	1.01 (1.007–1.012)^***^	1.01 (1.005–1.01) ^***^	1.001 (0.998–1.005)	–	1.01 (1.004–1.01)^***^	1.02 (1.01–1.02)^***^
*Men*						
NC	1.13 (1.06–1.21)^***^	–	1.05 (0.97–1.13)	1.09 (1.02–1.17)^*^	1.1 (1.01–1.2)^*^	1.11 (1.04–1.19)^**^
FPG	2.11 (1.86–2.41)^***^	2.08 (1.82–2.37)^***^	–	2.14 (1.86–2.46)^***^	2.1 (1.84–2.4)^***^	2.13 (1.86–2.43)^***^
LAP	1.01 (1.004–1.014)^***^	1.006 (1.001–1.011)^*^	0.998 (0.993–1.004)	–	1.007 (1.002–1.012)^**^	1.013 (1.001–1.025)^*^
*Women*						
NC	1.22 (1.15–1.29)^***^	–	1.14 (1.06–1.22)^***^	1.18 (1.11–1.26)^***^	1.2 (1.11–1.3)^***^	1.21 (1.14–1.28)^***^
FPG	2.3 (2.015–2.63)^***^	2.17 (1.91–2.48) ^***^	–	2.25 (1.96–2.57)^***^	2.23 (1.95–2.55)^***^	2.29 (2.004–2.62)^***^
LAP	1.01 (1.006–1.014)^***^	1.01 (1.001–1.009) ^*^	1.003 (0.99–1.008)	–	1.007 (1.003–1.011)^***^	1.02 (1.01–1.03)^***^

Notes^*^:*P* < 0.05;^**^:*P* < 0.01 ^***^:*P* < 0.001。Confounding factors are in the top row and risk factors of T2DM in the left column. LAP, Lipid Accumulation Product; NC: Neck circumference; FPG: Fasting Plasma Glucose; †: univariate analysis; ∫ multivariate analysis

**Table 7:** T2DM predicting effect of different indices

Variables	Cut-off value	Sensitivity (%)	Specificity (%)	Youden index	AUC (95%CI)	Model Quality	P value
Overall							
LAP	33.8	72%	44%	0.159	0.613 (0.581–0.645)	0.58	< 0.001^***^
NC	35.75	67.6%	48.7%	0.16	0.6 (0.569 − 0.532)	0.57	< 0.001^***^
FPG	6.4	75%	84%	0.592	0.839 (0.812–0.866)	0.81	< 0.001^***^
Male							
LAP	37.95	58%	59%	0.17	0.604 (0.577–652)	0.56	< 0.001^***^
NC	36.9	80.7%	43.7%	0.144	0.597 (0.594–0.644)	0.55	< 0.001^***^
FPG	6.49	72.7%	83.6%	0.563	0.835 (0.795–0.874)	0.79	< 0.001^***^
Female							
LAP	60.2	**45.4%**	71.8%	0.172	0.617 (0.574–0.660)	0.57	< 0.001^***^
FPG	6.42	77.6%	84.6%	0.622	0.842 (0.805–0.878)	0.81	< 0.001^***^

*Notes* AUC, area under the curve; LAP, lipid accumulation product; NC: Neck circumference; FPG: fasting plasma glucose; BMI: Body mass index; *, *P* < 0.05; ***, *P* < 0.001

## Data Availability

The raw data for producing this manuscript is available from the corresponding author upon reasonable request.
